# Long-term efficacy of lobectomy for stage T1 papillary thyroid carcinoma with varying degrees of lymph node metastasis

**DOI:** 10.3389/fendo.2024.1453601

**Published:** 2024-08-08

**Authors:** Chao Qin, Sijia Cai, Yanyu Qi, Meilin Liu, Weibo Xu, Min Yin, Haitao Tang, Qinghai Ji, Tian Liao, Yu Wang

**Affiliations:** ^1^ Department of Head and Neck Surgery, Fudan University Shanghai Cancer Center, Shanghai, China; ^2^ Department of Oncology, Shanghai Medical College, Fudan University, Shanghai, China; ^3^ Shanghai Medical College, Fudan University, Shanghai, China

**Keywords:** papillary thyroid carcinoma, lymph node metastasis, lymph node positive rate, N stage, recurrence

## Abstract

**Background:**

The presence of lymph node metastasis (LNM) is frequently observed in papillary thyroid carcinoma (PTC), and most clinical guidelines recommend total thyroidectomy. However, the impact of LNM on specific types of locoregional recurrence remains uncertain, particularly for stage T1 PTC.

**Methods:**

The present retrospective cohort study enrolled patients diagnosed with stage T1 PTC between 2008 and 2015. Propensity score matching was performed in patients with lobectomy accompanied by varying degrees of LNM. Logistic regression analysis was performed to compare the effect of LNM on relapse types, and Kaplan-Meier method was utilized to calculate recurrence-free survival.

**Results:**

The study cohort comprised 2,785 patients who were followed up for an average duration of 69 months. After controlling follow-up time and potential prognostic factors, we include a total of 362 patients in each group. Recurrence rates in the N0, N1a, and N1b groups were found to be 2.5%, 9.7%, and 10.2% respectively. Notably, group N1a versus group N0 (*P*=0.803), N1b group versus N0 group (*P*=0.465), and group N1b versus group N1a (*P*=0.344) had no difference in residual thyroid recurrence. However, when considering lymph node recurrence, both N1a(*P*=0.003) and N1b(*P*=0.009) groups showed a higher risk than N0 group. In addition, there was no difference in lymph node recurrence between N1b group and N1a group (*P*=0.364), but positive lymph node (PLN) and lymph node positive rate (LNPR) demonstrated a strong discriminatory effect (*P*<0.001).

**Conclusion:**

Lobectomy may be more appropriate for patients with unilateral stage T1 PTC in the low LNPR group.

## Introduction

The increasing utilization of high-resolution ultrasound and the widespread adoption of thyroid core needle biopsy have contributed to a rise in the diagnosis of T1 stage papillary thyroid cancer (PTC), which has been identified as the primary factor driving the surge in thyroid cancer surgeries in recent years (1–3). In patients with T1 stage PTC, lymph node metastasis (LNM) is the primary risk factor for recurrence (4). However, the optimal surgical approach for these individuals has been a subject of controversy (5, 6). Multiple guidelines recommend total thyroidectomy in patients with multiple central or lateral cervical lymph node metastases, which is justified by the potential benefit of postoperative iodine ablation or thyroglobulin follow-up (7, 8). On the contrary, however, recent studies have demonstrated that lobectomy and total thyroidectomy exhibit comparable rates of recurrence-free survival (RFS) and disease-specific survival in patients diagnosed with PTC accompanied by lymph node metastasis (9, 10).

In fact, the significance of contralateral residual thyroidectomy for these patients remains uncertain. Although LNM is considered a high-risk factor for locoregional recurrence, its role in the staging system has been attenuated (11), and there is no consistent evidence regarding the preventive effect of iodine ablation therapy on locoregional recurrence (12, 13). Moreover, the impact of LNM on specific types of locoregional recurrence, particularly the influence of LNM severity on residual thyroid recurrence after unilateral stage T1 PTC, remains unclear. Therefore, establishing a reliable association between LNM severity and prognosis is necessary to effectively identify the risk of locoregional recurrence (14).

The present study employed propensity score matching (PSM) analysis to compare the impact of LNM on locoregional recurrence in patients with stage T1 PTC. This approach aims to comprehensively elucidate the influence of LNM severity on different types of recurrence and to explore prognostic indicators that can better reflect the extent of LNM.

## Materials and methods

### Study population

The retrospective cohort included 2785 patients with a mean follow-up of 69 months (range: 6-265 months). All patients underwent initial surgical treatment at the Department of Head and Neck Surgery, Affiliated Cancer Hospital of Fudan University between January 1, 2008, and December 31, 2015. This retrospective study was approved by the Ethics Committee of the Affiliated Cancer Hospital of Fudan University (050432-4-2108), and informed consent was obtained at the time of surgery for the general use of clinical information for future studies.

Demographic, clinical, and pathological data were retrospectively collected using a standardized database template for all patients. The study enrolled patients diagnosed with stage T1 PTC who underwent standard unilateral lobectomy and central lymph node dissection, as well as patients with cN1b who underwent lateral lymph node dissection. Additionally, patients who had a contralateral benign nodule excised were also included. The exclusion criteria encompassed previous neck surgery, radiotherapy, metastatic lymph node exceeding 3cm, obvious microscopic extranodular extension, distant metastasis, second primary tumor, highly aggressive histological subtypes, contralateral thyroid malignancy tumor, and lymph node skip metastasis. Additionally, cases of re-operation within 6 months following the initial procedure were excluded to prevent residual effects.

All patients received postoperative conventional thyroid-stimulating hormone suppression, which was primarily determined by the surgeon. Postoperative follow-up adhered to the recommended strategies outlined in the 2015 American Thyroid Association Guidelines and the 8th edition American Joint Committee on Cancer (AJCC) Staging System Guidelines. The primary endpoint of the study was focused on locoregional structural recurrence, which was confirmed by both cytology and histopathology. Follow-up was conducted through medical records and telephone communication. The recurrent cases were categorized into three subtypes: residual thyroid involvement, cervical lymph node involvement, or a combination of both. The determination of the recurrence type was primarily based on medical records, preoperative ultrasound and enhanced CT scans, as well as postoperative pathological reports. Pathological diagnosis, including lymph node count, was conducted by two specialized head and neck pathologist.

### Study design

According to the 8th edition AJCC TNM-N classification system, the enrolled patients were categorized into N0, N1a, and N1b groups based on their pathological N stage. The primary objective of this study was to assess the impact of LNM on the specific patterns of locoregional recurrence. In order to enhance the reliability and comparability across groups, we employed a 1:1:1 PSM approach to balance variables that influence prognosis. Considering data characteristics and existing literature, we ultimately included age, gender, T stage, extrathyroidal extension, Hashimoto’s thyroiditis, multifocality, and follow-up time in the matching process. According to the 8th edition of AJCC guidelines, patients were categorized into two groups based on age (<55 and≥55 years), tumor size (T1a and T1b), while the diagnosis of thyroid capsule invasion and Hashimoto’s thyroiditis was determined through pathology rather than preoperative imaging or serology. Multifocal disease was defined as the presence of two or more isolated malignant lesions in a unilateral thyroid lobe. Furthermore, to underscore the prognostic significance of LNM, we conducted a comparative analysis of positive lymph node (PLN) and lymph node positive ratio (LNPR) as potential indicators for recurrence. LNPR denotes the ratio between the total count of metastatic nodes and the overall number of resected nodes.

### Statistical analysis

The sample setting and data collection methods in this study were consistent with the Enhanced Observational Epidemiological Study report statement. Continuous variables were presented as mean and standard deviation, while categorical variables were expressed as integers and percentages. Categorical variables were analyzed using Pearson χ2 test or Fisher exact test, meanwhile continuous variables were assessed using t-test or Wilcoxon rank sum test. Additionally, odds ratio (OR) of 95% confidence interval (CI) was precisely calculated. PSM analysis was employed to eliminate differences in baseline data among the three groups of patients. The R-packet twang was employed for 1:1:1 matching, with a caliper value of 0.05, and non-substitute matching was selected; N stage was used as a standard of group classification, and potential prognostic factors such as age, sex, T stage, extrathyroidal extension, Hashimoto’s thyroiditis, multifocal disease and follow-up time served as matching indexes. The area under curve (AUC) of the receiver operating characteristic was calculated to determine optimal cut-off points for PLN and LNPR. Logistic regression analysis was performed after matching in order to compare the impact of different LNM parameters on recurrence types, while the Kaplan-Meier method was used to assess the RFS rate between groups. All reported *P* values were two-tailed, and statistical significance was defined as *P*< 0.05. Statistical analyses were conducted using R software version 4.1.3 and IBM SPSS Statistics version 27.0.

## Results

### Baseline data before and after PSM

A total of 2785 patients diagnosed with unilateral stage T1 PTC were included in the study, and the baseline data are presented in **Table 1**. Among them, there were 1327 cases (48.7%) classified as N0 stage, 986 cases (32.2%) classified as N1a stage, and 472 cases (19.1%) classified as N1b stage. The male to female ratio was approximately 1:3, with a dominance of female patients as many as 2110(75.8%). Additionally, there were 434 patients (15.6%) aged 55 years or older and a majority of patients at stage T1a comprising of 1948 individuals (69.9%). The mean number of PLN was calculated to be approximately 2.21 ± 3.82, while the mean LNPR was found to be around 0.26 ± 0.33. Furthermore, it was observed that within the studied patient cohort, only 47 individuals (1.7%) exhibited thyroid capsule invasion. Hashimoto’s thyroiditis was present in 529 patients (19.0%), and multifocal cases were identified in 338 patients (12.1%). The average follow-up duration for these patients amounted to about 69 months (range: 6-265 months), and during this period 167 cases (6.0%) experienced relapse. Relevant disparities were observed among the three groups in terms of gender distribution, age composition, T-stage classification, presence of thyroid capsule invasion, Hashimoto’s thyroiditis, multiple lesions, and follow-up duration.

**Table 1:** Demographic, clinical and pathological characteristics of patients by extent of lymph node metastasis.

Variables	Overall (%)	N0 (%)	N1a (%)	N1b (%)	*P*
	(N=2785)	(N=1327)	(N=986)	(N=472)	
Gender					<0.001
Female	2110(75.8)	1073 (80.9)	704 (71.4)	333 (70.6)	
Male	675(24.2)	254 (19.1)	282 (28.6)	139 (29.4)	
Age (year)					<0.001
<55	2351(84.4)	1080 (81.4)	863 (87.5)	408 (86.4)	
≥55	434(15.6)	247 (18.6)	123 (12.5)	64 (13.6)	
T stage					<0.001
T1a	1948(69.9)	1089 (82.1)	656 (66.5)	203 (43.0)	
T1b	837(30.1)	238 (17.9)	330 (33.5)	269 (57.0)	
PLN					<0.001
Mean (SD)	2.21(3.82)	0 (0)	2.40 (1.92)	8.04 (5.53)	
LNPR					<0.001
Mean (SD)	0.26(0.33)	0 (0)	0.59 (0.30)	0.30 (0.19)	
Capsular invasion					<0.001
Yes	47(1.7)	9 (0.7)	18 (1.8)	20 (4.2)	
No	2738(98.3)	1318 (99.3)	968 (98.2)	452 (95.8)	
Hashimoto thyroiditis					<0.001
Yes	529(19.0)	294 (22.2)	165 (16.7)	70 (14.8)	
No	2256(81.0)	1033 (77.8)	821 (83.3)	402 (85.2)	
Multi-focus					<0.001
Yes	338(12.1)	135 (10.2)	121 (12.3)	82 (17.4)	
No	2447(87.9)	1192 (89.8)	865 (87.7)	390 (82.6)	
Follow-up (month)					<0.001
Mean (SD)	69.25(44.12)	67.01 (39.06)	66.49 (48.27)	81.31(46.52)	
Follow-up (state)					<0.001
Recurrence	167(6.0)	29 (2.2)	85 (8.6)	53 (11.2)	
RT	51(30.54)	23(79.31)	24(28.24)	4(7.55)	
LN	66(39.52)	4(13.79)	42(49.41)	20(37.74)	
RT+LN	50(29.94)	2(6.90)	19(22.35)	29(54.72)	
No recurrence	2618(94.0)	1298 (97.8)	901 (91.4)	419 (88.8)	

PLN, positive lymph node; LNPR, lymph node positive ratio; RT, residual thyroid; LN, lymph node.

After conducting PSM, any disparities in prognostic factors, such as age, sex, T stage, thyroid capsule invasion, Hashimoto’s thyroiditis, multifocality and follow-up time were effectively mitigated. Finally, a total of 1086 patients were included for subsequent analysis (**Table 2**). The mean number of PLN after matching was 3.47, while the average LNPR stood at 0.29. In stage N0, thyroid residual recurrence occurred in seven cases; one case had lymph node recurrence and one case had both recurrences. For stage N1a, there were seven cases of thyroid residue recurrence; nineteen cases with lymph node recurrence and nine cases with both recurrences. As for stage N1b, four cases had thyroid residue recurrence; fourteen cases had lymph node recurrence and nineteen cases had both recurrences.

**Table 2:** Demographic, clinical and pathological characteristics of patients by extent of lymph node metastasis after PSM.

Variables	Overall (%)	N0 (%)	N1a (%)	N1b (%)	*P*
	(N=1086)	(N=362)	(N=362)	(N=362)	
Gender					0.098
Female	791(72.8)	278 (76.8)	260 (71.8)	253 (69.9)	
Male	295(27.2)	84 (23.2)	102 (28.2)	109 (30.1)	
Age (year)					0.993
<55	928(85.5)	309 (85.4)	309 (85.4)	310 (85.6)	
≥55	158(14.5)	53 (14.6)	53 (14.6)	52 (14.4)	
T stage					0.932
T1a	508(46.8)	167 (46.1)	169 (46.7)	172 (47.5)	
T1b	578(53.2)	195 (53.9)	193 (53.3)	190 (52.5)	
PLN					<0.001
Mean (SD)	3.47(4.87)	0 (0)	2.48 (2.10)	7.91 (5.84)	
LNPR					<0.001
Mean (SD)	0.29(0.32)	0 (0)	0.58 (0.31)	0.29 (0.19)	
Capsular invasion					0.326
Yes	36(3.3)	8 (2.2)	13 (3.6)	15 (4.1)	
No	1050(96.7)	354 (97.8)	349 (96.4)	347 (95.9)	
Hashimoto thyroiditis					0.864
Yes	171(15.7)	54 (14.9)	59 (16.3)	58 (16.0)	
No	915(84.3)	308 (85.1)	303 (83.7)	304 (84.0)	
Multi-focus					0.514
Yes	165(15.2)	56 (15.5)	49 (13.5)	60 (16.6)	
No	921(84.8)	306 (84.5)	313 (86.5)	302 (83.4)	
Follow-up (month)					0.917
Mean (SD)	69.18(38.35)	69.43(38.01)	68.51(39.07)	69.61 (38.05)	
Follow-up (state)					<0.001
Recurrence	81(7.5)	9 (2.5)	35 (9.7)	37 (10.2)	
RT	18(22.22)	7(78.77)	7(20.00)	4(10.81)	
LN	34(41.98)	1(11.11)	19(54.29)	14(37.84)	
RT+LN	29(35.80)	1(11.11)	9(25.71)	19(51.35)	
No recurrence	1005(92.5)	353 (97.5)	327 (90.3)	325 (89.8)	

PLN, positive lymph node; LNPR, lymph node positive ratio; RT, residual thyroid; LN, lymph node.

### The influence of N stage on locoregional recurrence patterns

In terms of residual thyroid recurrence, the recurrence rate did not increase with the increase of N stage, even when controlling factors including age, sex, T stage, capsule invasion, Hashimoto’s thyroiditis, and multiple focal lesions. Specifically, there was no significant increase in recurrence for N1a (OR=1.15; *P*=0.803) or N1b (OR=0.63; *P*=0.465) cases. In terms of lymph node recurrence, N1a (OR=20.51, 95% CI: 4.22-369.57, *P*=0.003) and N1b (OR=15.21, 95% CI: 3.03-276.34, *P*=0.009) exhibited a higher risk of recurrence; however, even after controlling associated risk factors, there was no significant difference in the risk of recurrence between N1a and N1b. This trend was also observed in residual thyroid combined with lymph node recurrence (**Table 3**). Thus, these findings suggest that the presence of LNM is irrelevant with the risk of residual thyroid recurrence.

**Table 3:** Effect of N stage on cervical recurrence type of stage T1 papillary thyroid carcinoma.

Recurrence	N stage	Unadjusted		Adjusted^a^	
type		OR (95%CI)	*P*	OR (95%CI)	*P*
RT					
	N1a vs. N0	1.08(0.37-3.19)	0.883	1.15(0.38-3.42)	0.803
	N1b vs. N0	0.62(0.16-2.08)	0.453	0.63(0.16-2.12)	0.465
	N1b vs. N1a	0.58(0.15-1.92)	0.381	0.55(0.14-1.85)	0.344
LN					
	N1a vs. N0	20.51(4.22-369.57)	0.003	21.39(4.38-386.24)	0.003
	N1b vs. N0	15.21(3.03-276.34)	0.009	15.38(3.05-279.96)	0.009
	N1b vs. N1a	0.74(0.36-1.50)	0.407	0.41(0.35-1.46)	0.364
RT+LN					
	N1a vs. N0	9.74(1.82-180.17)	0.031	9.60(1.78-177.79)	0.033
	N1b vs. N0	20.70(4.26-372.90)	0.003	20.04(4.10-361.82)	0.004
	N1b vs. N1a	2.12(0.97-5.00)	0.068	2.09(0.95-4.94)	0.076

RT, residual thyroid; LN, lymph node;

a Adjusted for gender, age, T stage, Capsular invasion, Hashimoto thyroiditis and Multifocal.

### The influence of PLN and LNPR risk stratification on locoregional recurrence patterns

PLN and LNPR have been reported to provide a more accurate assessment of LNM severity and are associated with locoregional recurrence. Therefore, we conducted further investigations to explore the connection between risk stratification(measured by PLN and LNPR) and recurrence patterns. Firstly, the cutoff values for PLN and LNPR were determined using receiver operating characteristic curve analysis, with values of 3 (AUC=0.686) and 0.42 (AUC=0.706), respectively. Based on these values, patients were categorized into high-risk and low-risk groups. There was no significant increase in residual thyroid recurrence observed in the high PLN (OR=1.75; 95%CI: 0.64-5.54; *P*=0.299) or high LNPR (OR=0.70; 95%CI: 0.27-2.07; *P*=0.492) groups when compared to their respective low-risk counterparts, even when controlling age, sex, T stage, capsule invasion, Hashimoto’s thyroiditis, and multiple focal lesions. Significant differences were observed between risk groups defined by PLN and LNPR regarding lymph node recurrence as well as both recurrences combined (*P*<0.001) (**Table 4**). Thus, the above result offers additional evidence that the presence of severe LNM does not pose a risk for residual thyroid recurrence.

**Table 4:** Effect of PLN and LNPR risk stratification on cervical recurrence types of stage T1 papillary thyroid carcinoma.

Recurrence	Variables	Unadjusted		Adjusted^a^	
type		OR (95%CI)	*P*	OR (95%CI)	*P*
RT					
	HPLN vs. LPLN	0.63(0.20-1.69)	0.388	1.75(0.64-5.54)	0.299
LN					
	HPLN vs. LPLN	0.25(0.12-0.52)	<0.001	0.27(0.12-0.55)	<0.001
RT+LN					
	HPLN vs. LPLN	0.10(0.03-0.25)	<0.001	0.10(0.03-27.00)	<0.001
RT					
	HLNPR vs. LLNPR	1.42(0.49-3.69)	0.489	0.70(0.27-2.07)	0.492
LN					
	HLNPR vs. LLNPR	0.19(0.09-0.39)	<0.001	0.19(0.09-0.40)	<0.001
RT+LN					
	HLNPR vs. LLNPR	0.16(0.07-0.34)	<0.001	0.17(0.07-37.30)	<0.001

RT, residual thyroid; LN, lymph node; HPLN, high positive lymph node; LPLN, low positive lymph node; HLNPR, high lymph node positive ratio; LLNPR, low lymph node positive ratio;

a Adjusted for gender, age, T stage, Capsular invasion, Hashimoto thyroiditis and Multifocal.

### Recurrence-free survival analysis after PSM

**Figure 1:**
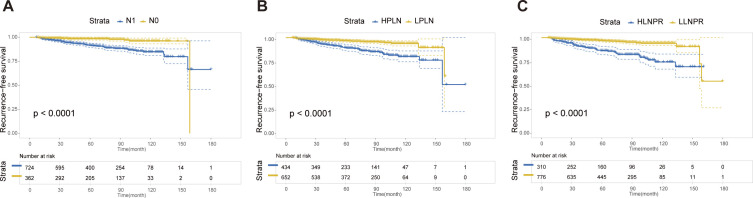
The RFS Kaplan-Meier curves for overall locoregional recurrence according to N Stage **(A)**, PLN **(B)**, and LNPR **(C)** risk stratification after PSM in the enrolled patients. RFS, recurrence-free survival; PSM, propensity score matching; HPLN, high positive lymph node; LPLN, low positive lymph node; HLNPR, high lymph node positive ratio; LLNPR, low lymph node positive ratio.

**Figure 2:**
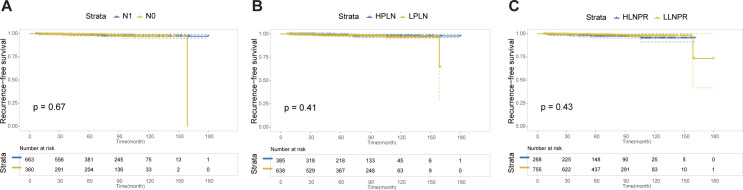
The RFS Kaplan-Meier curves for residual thyroid recurrence according to N Stage **(A)**, PLN **(B)**, and LNPR **(C)** risk stratification after PSM in the enrolled patients. RFS: recurrence-free survival, PSM: propensity score matching, HPLN, high positive lymph node; LPLN, low positive lymph node; HLNPR, high lymph node positive ratio; LLNPR, low lymph node positive ratio.

**Figure 3:**
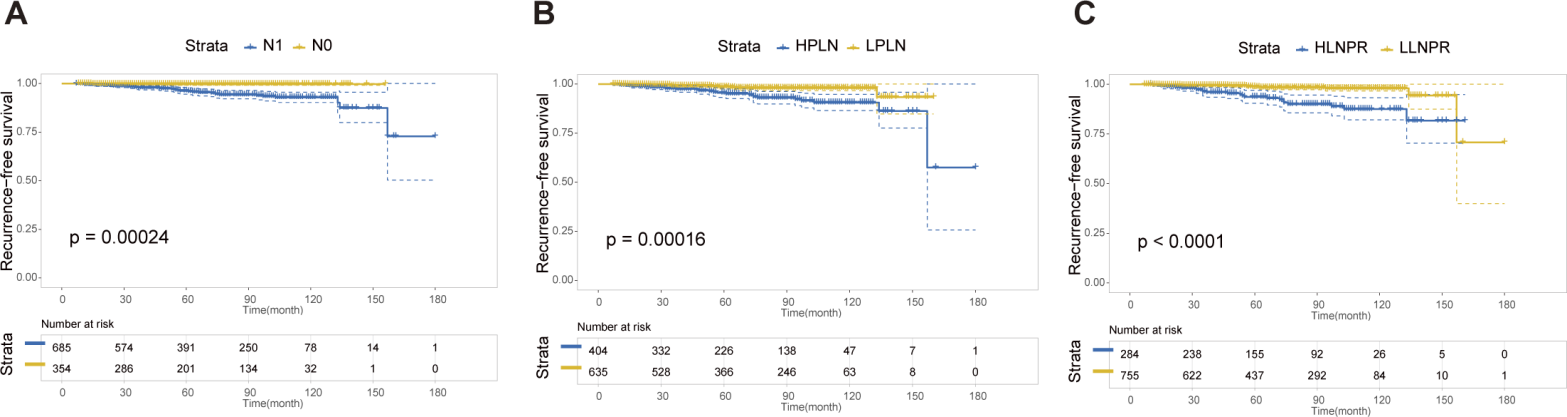
The RFS Kaplan-Meier curves for cervical lymph node recurrence according to N Stage **(A)**, PLN **(B)**, and LNPR **(C)** risk stratification after PSM in the enrolled patients. RFS, recurrence-free survival; PSM, propensity score matching; HPLN, high positive lymph node; LPLN, low positive lymph node; HLNPR, high lymph node positive ratio; LLNPR, low lymph node positive ratio.

**Figure 4:**
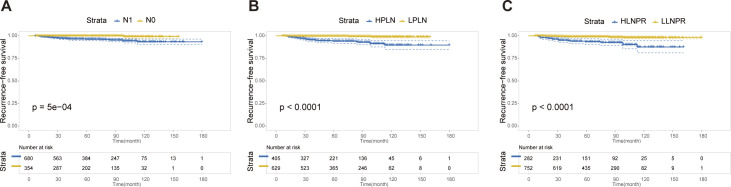
The RFS Kaplan-Meier curves for both residual thyroid and cervical lymph node recurrence according to N Stage **(A)**, PLN **(B)**, and LNPR **(C)** risk stratification after PSM in the enrolled patients. RFS, recurrence-free survival; PSM, propensity score matching; HPLN, high positive lymph node; LPLN, low positive lymph node; HLNPR, high lymph node positive ratio; LLNPR, low lymph node positive ratio.

After PSM, the average duration of follow-up for the N0, N1a, and N1b groups was 69.43, 68.51 and 69.61 months, respectively. The respective 5-year RFS rates were 96.8%, 85.2% and 84.8%. Next, we compared the differences in RFS among patients stratified by stage N, PLN, and LNPR risk classifications. In terms of overall locoregional recurrence, all three measures effectively distinguish high-risk patients (*P*<0.001) (**Figure 1**). However, when it comes to residual thyroid recurrence, the three classifications (N stage, *P*=0.67; PLN, *P*=0.41; LNPR, *P*=0.43) do not provide effective discrimination (**Figure 2**). For lymph node recurrence (**Figure 3**) or a combination of both types of recurrences (**Figure 4**), all three parameters demonstrate superior predictive ability, with LNPR being the most reliable predictor (*P*<0.001).

## Discussion

In this cohort study, we investigated the impact of LNM on different types of locoregional recurrence in patients with unilateral stage T1 PTC. To establish a comparable cohort, we enrolled patients with varying degrees of cervical LNM while ensuring similar tumor size and surgical scope. PSM analysis was conducted to minimize the influence of relevant prognostic factors. The findings demonstrated that residual thyroid recurrence in patients with unilateral T1 PTC was not influenced by N stage. However, as N stage advanced, there was a significant increase in the rate of cervical lymph node recurrence. Additionally, we have established prognostic predictors PLN and LNPR, which are effective predictors of locoregional recurrence. This can assist clinicians in formulating targeted treatment strategies and implementing follow-up programs.

Due to the widespread usage of prophylactic central lymph node dissection, the diagnosis rate of LNM is increasing in patients with stage T1 PTC (15). This not only facilitates accurate diagnosis but also assists surgeons in formulating evidence-based treatment plans. LNM has been identified as a significant risk factor for locoregional recurrence based on numerous previous studies (16, 17). Therefore, most guidelines recommend total thyroidectomy for cases with LNM, particularly those at high risk of recurrence (18, 19). In clinical practice, the primary objective of contralateral thyroidectomy is to facilitate iodine ablation, prevent recurrence, and monitor thyroglobulin levels; however, its utility in low-risk PTC remains uncertain (20). Consequently, it is crucial to elucidate the impact of LNM on residual thyroid recurrence, and this study fills that research gap. According to the findings of this study, in cases of unilateral T1 stage PTC, it is safe to preserve the contralateral healthy thyroid gland after excluding other risk factors. This approach is justified by the fact that LNM does not impact the residual thyroid in terms of recurrence. In such scenarios, a more precise extent of lymph node dissection or vigilant follow-up should be employed. Ultimately, most patients exhibit favorable prognoses even if they undergo multiple surgeries. To summarize, when discussing the influence of LNM on locoregional recurrence, it is essential to comprehensively consider variations in risk factors and different types of recurrence.

In recent years, the concept of LNM has gained significant attention in surgical decision-making, particularly for patients with N1b disease. Due to the compelling evidence of favorable long-term prognosis, an increasing number of surgeons are now inclined towards preserving healthy thyroid (21, 22). Xu et al. employed PSM analysis to investigate the disparity between unilateral thyroidectomy and total thyroidectomy in N1b patients, revealing that there was no statistically significant difference in RFS between the two cohorts (23). Additionally, a retrospective study conducted by Memorial Sloan-Kettering Cancer Center demonstrated that following appropriate surgical intervention, the majority of patients with low-risk PTC, including some individuals with advanced tumors or regional metastases, do not necessitate iodine ablation and exhibit a low recurrence rate along with high survival rates (24). Although patients in the N1b group in our study exhibited a slightly higher overall recurrence rate, they still achieved a comparable RFS to those in the N1a group. Hence, it is imperative to reassess the rationale behind total thyroidectomy and to emphasize the importance of individualized treatment, in order to prevent overtreatment.

Furthermore, in the case of T1 PTC, LNM often emerges as the predominant factor contributing to recurrence. Our findings demonstrate that the N stage does not serve as a reliable prognostic indicator since there is no discernible advantage of N1b over N1a in distinguishing locoregional recurrence. This raises the question of whether LNM region (N1b) or ratio of metastatic lymph nodes more accurately reflects the disease state. Instead, PLN and LNPR prove to be more indicative of the severity of LNM and are widely employed as prognostic indicators across various cancers (25–27). The primary hindrance to their widespread application is standardization issues, including varied preferences of sample selection, procession, and statistical approaches. Nevertheless, it is undeniable that these indicators offer valuable insights for accurately predicting locoregional recurrence in patients with PTC.

While efforts were made to mitigate the impact of confounding factors on prognosis, the retrospective nature of this study may compromise the consistency of conclusions. Firstly, it should be acknowledged that multiple surgeons are often involved in one cancer center, rendering to varied degrees of neck lymph node dissection. Secondly, although severe extranodal invasion has been excluded, distinguishing between lymph nodes with micro-metastases and those with macro- transfer remains challenging. Thirdly, due to limited availability of molecular tests during this period, identification of gene mutations such as BRAF V600E or TERT promoters was not possible from the database. Additionally, cases involving unilateral lymph node dissection were included in this study, which may potentially yield a higher LNPR value (28, 29). Nevertheless, this study provides a robust theoretical foundation for future multicenter prospective studies.

## Conclusions

In conclusion, the findings of this single-institution study suggest that unilateral stage T1 PTC with cervical LNM is irrelevant with residual thyroid recurrence when controlling for major prognostic factors. Therefore, careful consideration should be given to the necessity of total thyroidectomy in patients with unilateral stage T1 PTC. Additionally, our results demonstrate that both PLN and LNPR can effectively identify patients at high risk of relapse following surgery for PTC.

## Data availability statement

The original contributions presented in the study are included in the article/supplementary material. Further inquiries can be directed to the corresponding authors.

## Ethics statement

The studies involving humans were approved by The Ethics Committee of the Affiliated Cancer Hospital of Fudan University. The studies were conducted in accordance with the local legislation and institutional requirements. The participants provided their written informed consent to participate in this study.

## Author contributions

CQ: Writing – original draft, Methodology, Formal analysis, Conceptualization. SC: Writing – original draft, Methodology, Formal analysis, Conceptualization. YQ: Writing – review & editing, Investigation. ML: Writing – review & editing, Investigation. WX: Writing – review & editing, Formal analysis. MY: Writing – review & editing, Formal analysis. HT: Writing – review & editing, Formal analysis. QJ: Writing – review & editing. TL: Writing – review & editing, Writing – original draft, Conceptualization. YW: Writing – review & editing, Writing – original draft, Funding acquisition, Conceptualization.
